# Radiomics and visual analysis for predicting success of transplantation of heterotopic glioblastoma in mice with MRI

**DOI:** 10.1007/s11060-024-04725-z

**Published:** 2024-07-03

**Authors:** Sabine Wagner, Christian Ewald, Diana Freitag, Karl-Heinz Herrmann, Arend Koch, Johannes Bauer, Thomas J. Vogl, André Kemmling, Hubert Gufler

**Affiliations:** 1 Department of Neuroradiology, Marburg University Hospital – Philipps University, 35043 Marburg, Germany; 2 Department of Neuroradiology, Institute for Diagnostic and Interventional Radiology, Jena University Hospital - Friedrich Schiller University, 07747 Jena, Germany; 3 Department of Neurosurgery, Brandenburg Medical School, Theodor Fontane, University Hospital Brandenburg/Havel, 14770 Brandenburg/Havel, Germany; 4 Department of Neurosurgery, Section of Experimental Neurooncology, Jena University Hospital - Friedrich Schiller University, 07747 Jena, Germany; 5 Medical Physics Group, Institute for Diagnostic and Interventional Radiology, Jena University Hospital - Friedrich Schiller University, 07743 Jena, Germany; 6 Department of Neuropathology, Charité - Universitätsmedizin Berlin, corporate member of Freie Universität Berlin, and Berlin Institute of Health, Charité University Medicine, 10117 Berlin, Germany; 7 Department of Diagnostic and Interventional Radiology, Goethe University Hospital Frankfurt, 60590 Frankfurt Am Main, Germany

**Keywords:** Magnetic resonance imaging, Glioblastoma, Tumor cell proliferation, Experimental study, Radiomic feature analysis

## Abstract

**Background:**

Quantifying tumor growth and treatment response noninvasively poses a challenge to all experimental tumor models. The aim of our study was, to assess the value of quantitative and visual examination and radiomic feature analysis of high-resolution MR images of heterotopic glioblastoma xenografts in mice to determine tumor cell proliferation (TCP).

**Methods:**

Human glioblastoma cells were injected subcutaneously into both flanks of immunodeficient mice and followed up on a 3 T MR scanner. Volumes and signal intensities were calculated. Visual assessment of the internal tumor structure was based on a scoring system. Radiomic feature analysis was performed using MaZda software. The results were correlated with histopathology and immunochemistry.

**Results:**

21 tumors in 14 animals were analyzed. The volumes of xenografts with high TCP (H-TCP) increased, whereas those with low TCP (L-TCP) or no TCP (N-TCP) continued to decrease over time (*p* < 0.05). A low intensity rim (rim sign) on unenhanced T1-weighted images provided the highest diagnostic accuracy at visual analysis for assessing H-TCP (*p* < 0.05). Applying radiomic feature analysis, wavelet transform parameters were best for distinguishing between H-TCP and L-TCP / N-TCP (*p* < 0.05).

**Conclusion:**

Visual and radiomic feature analysis of the internal structure of heterotopically implanted glioblastomas provide reproducible and quantifiable results to predict the success of transplantation.

**Supplementary Information:**

The online version contains supplementary material available at 10.1007/s11060-024-04725-z.

## Introduction

Magnetic resonance imaging (MRI) represents the noninvasive gold standard method for diagnosing, planning the treatment, and monitoring the therapy of brain tumors [1, 2]. MRI is also gaining increasing importance for studying tumor growth and treatment response in experimental models in small animals in vivo [3]. Using MRI to noninvasively study animals over a prolonged course of disease development and treatment would eliminate the need to sacrifice similarly sized groups of animals at each given time point during a study, thereby reducing the number of animals required [4, 5].

In order to understand brain tumor biology and to develop new treatment strategies, several experimental animal models of glial tumors have been developed. The human tumor xenograft is the most widely used model: here, human glioblastoma multiforme cells are transplanted orthotopically into the brain or, less frequently, heterotopically into the subcutaneous tissue of immunocompromised mice [6].

However, quantifying tumor growth and treatment response noninvasively poses a challenge to all tumor models [7]. Tumor growth is not necessarily characterized best by increased lesion size; indeed, additional criteria addressing changes in lesion density, homogeneity, vascularization, solid components, necrosis, and intratumoral hemorrhage may be important as well [8]. The difficulty in following the tumor growth of subcutaneous tumor models only by means of caliper measurements lies in the low reproducibility and sensitivity with regard to small volume fluctuations. Ideally, the standardized, quantifiable, and, thus, objectively comparable tumor cell proliferation before and after treatment should form the basis of any therapy study.

Detailed anatomic features can be visualized with MRI due to its excellent soft-tissue image contrast. An isotropic spatial resolution of (0.33 mm)^3^ can be achieved even on clinical scanners within an acceptable scan time, rendering longitudinal, structural, and morphological investigations of rodents feasible [9]. Radiomic feature analysis is a method by which mathematically defined features can be extracted from diverse medical images and provide quantitative information beyond what the human eye can assess [10]. In previous studies, radiomic feature analysis was applied as a radiological tool for differentiation, treatment monitoring, and prognosis prediction of various types of tumors [11–17].

In this study, we tested the usefulness of quantitative, qualitative-visual, and automated radiomic feature analysis of the interior tumor structure based on high-resolution MRI to predict the success of heterotopically transplanted glioblastoma in mice at the earliest possible stage in serial imaging.

## Materials and methods

Animal interventions and MRI scans were performed in accordance with the European Convention for Animal Care and Use of Laboratory Animals and were approved by the THÜRINGER LANDESAMT FÜR VERBRAUCHERSCHUTZ (TVL 02–029/13).

### Animal setup

A172 human glioblastoma cells [18] were cultured in DMEM (Gibco® DMEM, Darmstadt, Germany) supplemented with 10% fetal bovine serum. Cells were routinely passed with trypsin (0.05%)/ EDTA. For xenotransplantation the cells were separated with trypsin (0.05%)/ EDTA for 5 min, washed with 1 × DPBS (GIBCO® DPBS, Darmstadt, Germany), and adjusted to 1 × 10^7^ cells/ml.

We used immunodeficient mice (NOD.CB17-Prkdc^scid^/NCrHsd, Harlan Laboratories, Rossdorf, Germany) maintained under specific pathogen-free conditions. The xenografts were established in 8- to 10-week-old female animals by subcutaneously injecting 1 × 10^6^ A172 cells into both groins. A single dose consisted of 100 µl cell suspension mixed with 100 µl growth factor-reduced matrigel (BD Matrigel™ Basement Membrane Matrix, BD Biosciences, Erembodegem, Belgium). Anaesthesia was provided by an isoflurane vaporizer placed outside the radiofrequency cabin of the scanner. A lateral tail vein was cannulated in order to remotely administer Gd- DTPA (Gadovist®, Bayer Vital GmbH, Leverkusen, Germany). Respiration and heart rate were monitored using a pressure pad that was placed under the thorax close to the heart of the animal. To prevent hypothermia during imaging, the area surrounding the animals was heated. Rectal temperature was monitored using the FLUOTEMP® fiber optic temperature sensor and converter system (Photon Control Inc, Burnaby BC, Canada).

### MRI examination and analysis

All studies were performed on a clinical whole-body 3 T scanner (Magnetom TIM Trio, Siemens Healthcare, Erlangen, Germany) using a dedicated transmit-receive coil with a diameter of 35 mm and a linearly polarized Litz volume resonator design (Doty Scientific Inc., Columbia, SC, USA). The imaging protocol was standardized for timing and sequence order. A 3D T2-weighted (T2w SPACE) sequence with fat saturation and a voxel size of 0.26 × 0.26 × 0.25 mm was applied, followed by a 3D T1-weighted (T1w VIBE) sequence before and after intravenously administering Gd-DTPA (0.1 mmol/kg) with a voxel size of 0.24 × 0.24 × 0.25 mm. The time needed to acquire all imaging data of the MRI protocol was typically 40 min per mouse.

The MRI data were analyzed using the software tool XrayLine™ Workstation 2.0. Qualitative and quantitative analyses were carried out on coronary reconstructions of the 3D volume data sets.

Qualitative assessment of each MR measurement was adapted from the VASARI (Visually AcceSAble Rembrandt Images) MRI feature set [19]: the contrast-enhancement quality; the proportion of enhancing portions; the proportion of necrosis; the signal intensity (SI) of the rim in T1w precontrast images; the thickness and definition of the enhancing margin; and the presence of cysts, hemorrhage, and feeding vessels (description of each feature used and overview of the results of visual qualitative analysis are provided in the supplement: Supplementary Table S1).

The quantitative longitudinal analysis included measurements of the volume, the SI, the radiographic heterogeneity index (HI) of the lesion, and the areas of enhancing and nonenhancing compartments. The volume of the entire lesion was assessed from the precontrast T1w images and calculated for every MRI examination according to Cavalieri’s method (total volume [mm^3^] = sum of all cut surface areas [mm^2^] x slice thickness [mm]) [20]. For further quantitative analysis the image with the largest cross-sectional diameter of the lesion was chosen. To determine the SI, a polygonal region of interest (ROI) was drawn, covering the entire lesion on T2w and on T1w pre– and postcontrast sequences. The mean value was normalized by calculating the quotient of the mean value of the lesion and the mean value of the neighboring muscle. Additionally, the standard deviation (SD) of each measurement was recorded. The HI of the tumor was calculated by measuring the SD of the SI of the tumor normalized to the SD of the SI of the neighboring muscle. In a next step, the areas of the contrast-enhancing and the noncontrast-enhancing parts of the lesion were measured by placing appropriate ROIs.

For automatic radiomic feature analysis, the image with the largest cross-sectional diameter of all lesions on every sequence of the last and penultimate MRI examination was saved and imported into the freely available MaZda software (Institute of Electronics, Technical University Lodz, Poland) (list of radiomic features provided by the MaZda software are listed in the supplement: Supplementary Table S2) [21–24]. ROIs were drawn on the images with the largest tumor area, in the training data set (penultimate MRI examination), and on the test data set (last MRI examination).

### Pathological analysis

After the last MRI examination, all mice were euthanized. The surgical specimens were evaluated by a neuropathologist. The tumors were fixed in 10% phosphate-buffered formalin, embedded in paraffin, serially sectioned at 3 µm, and stained with hematoxylin and eosin (H&E) for histological examination. Additionally, immunocytochemistry was used to assess glial fibrillary acidic protein (GFAP) and Ki67 was performed.

All tumors were divided into three groups according to tumor cell proliferation in the histological evaluation as follows: high tumor cell proliferation (H-TCP) (Ki67 ≥ 10%—50%), low tumor cell proliferation (L-TCP) (Ki67 < 10%), and no evidence of tumor cell proliferation (N-TCP). Tumor cell proliferation, necrotic parts, and vascular proliferations were evaluated and recorded.

The results of the qualitative and quantitative analysis of the MR images from the last MRI examination as well as the radiomic feature analysis of these images were compared to the histological findings.

### Statistical Analysis

Statistical analysis was performed using the open-source statistics package R (R Foundation for Statistical Computing, Vienna, Austria) [25] with RStudio 4.3.6 (packages: tidyverse, pROC, caret, janitor, Boruta, DescTools). For normally distributed data, the independent sample t-test was employed. ANOVA and Tukey–Kramer statistics with Benjamini–Hochberg correction for multiple testing was used with continuous parameters. Categorical variables derived from the qualitative analysis were compared between the tumor groups by applying the chi-squared test. The significance level was set at 0.05.

We did not use the feature selection tool provided by the MaZda software; instead, the results of the extracted features were transferred into the R software for further statistical analysis. Since the number of features derived from radiomic feature analysis was by far higher than the number of observations, a rigorous variable selection procedure was necessary. First, the intra- and interobserver reproducibility of all extracted radiomic features was assessed for the training data set, and all features with an intraclass correlation coefficient (ICC) of < 0.80 were excluded. Then, the remaining radiomic features were analyzed and selected using a machine learning algorithm (Boruta) to further reduce dimensions. The Boruta algorithm was used for feature selection applying 500 decision trees (tree depth 5 splits). The RandomForest classifier was used to obtain feature importance.

## Results

In 18 mice, tumor cells were implanted in both flanks. Four mice died during the observation period (two on day 4 after tumor cell implantation and one each on day 27 and 30). The tumors grew on both sides in 7 mice and only on one side in 7 mice. A total of 21 tumors in 14 animals were followed up longitudinally with MRI and the animals were euthanized after the last MRI examination for histological work-up. In summary, 85 MRI measurements were available for longitudinal data analysis: 3 measurements each for 3 tumors in 3 mice, 4 measurements each for 14 tumors in 9 mice, and 5 measurements each for 4 tumors in 2 mice. The first measurement took place between the 3rd and 12th day after cell implantation, all other measurements between the 18th and 54th, 56th and 84th, 88th and 147th days, and on the 175th day.

### Histology

Ten out of 21 tumors were solid, very densely cell-packed with high mitotic and proliferative activity (Ki67 ≥ 10% to 50%), showing necrosis (30%) and neovascular proliferation (50%). Five out of 21 tumors contained only a few sparse tumor cell groups showing low proliferative activity (Ki67 5% to < 10%). No tumor cells could be detected in 6 xenografts. Regarding the morphology, an astroglial (glioblastoma-typical) differentiation was evident (an example of successful tumor growth and histological work-up is given in the supplement: Supplementary Figure S3).

### MRI analysis

For data analysis, the 21 tumors were divided into two groups based on their histology: H-TCP tumors (48%), on the one hand, and L-TCP tumors or N-TCP tumors (52%), on the other. The results of the evaluation of the quantitative and qualitative MRI parameters for the two groups were tested pairwise for significant differences.

**Fig. 1:**
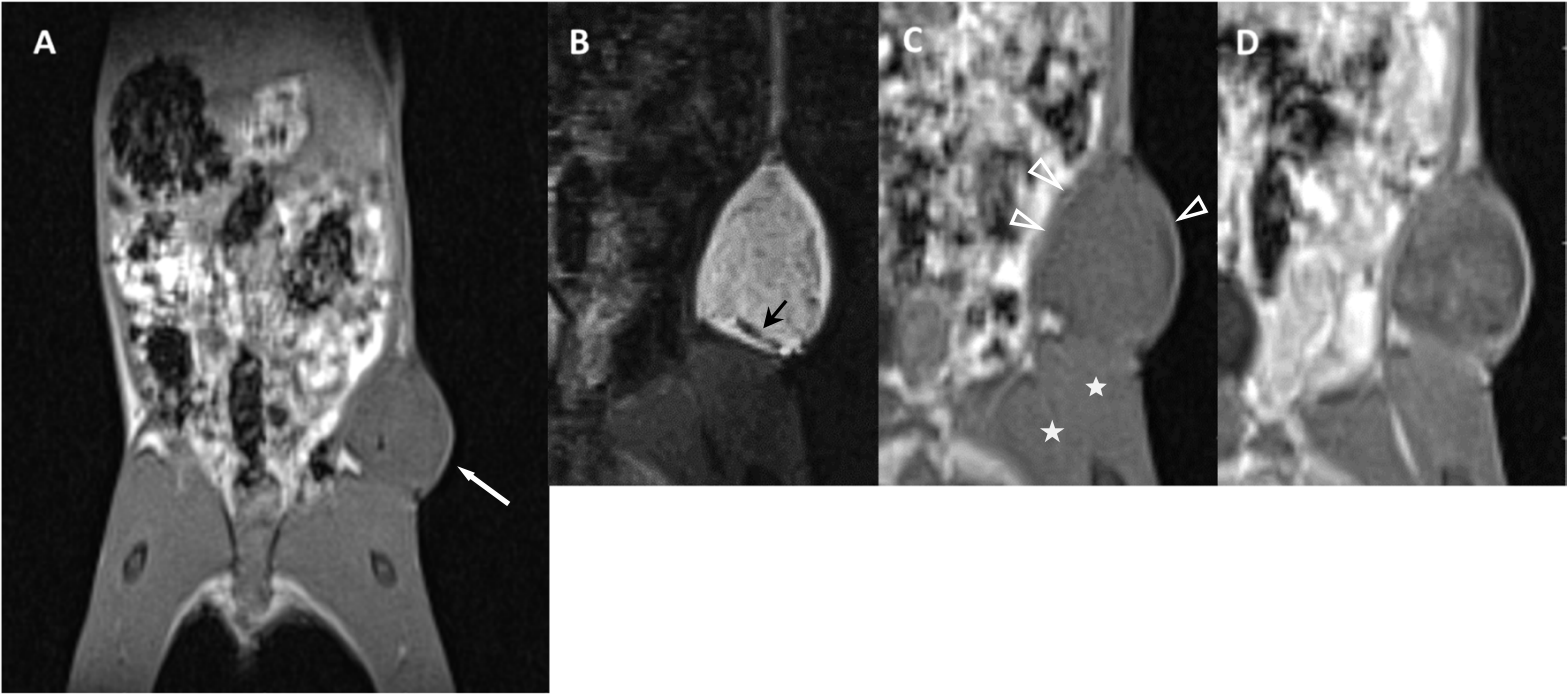
Successful tumor growth after implanting a xenograft in the left flank of an immunodeficient female mouse. **A** Coronal reconstruction from the 3D volume precontrast T1w MR-data set with a 0.3-mm slice thickness showing the abdomen, pelvis, and thighs of the mouse. In prone position the animal was fixed with stretched legs. The tumor in the right groin had a maximum diameter of 9.7 × 8.2 mm (white arrow). B—D A triplet of the magnified tumor is given. The T2w image **B** shows an inhomogeneous internal structure. Areas with linear signal voids (black arrow) within the tumor are seen as a correlate for histologically confirmed neovascular proliferation. T1w precontrast images **C** showing a rim (white arrowheads) isointense to the neighboring muscle (white asterix). Inhomogeneous enhancement of the tumor after administering of contrast agent **D**

**Fig. 2:**
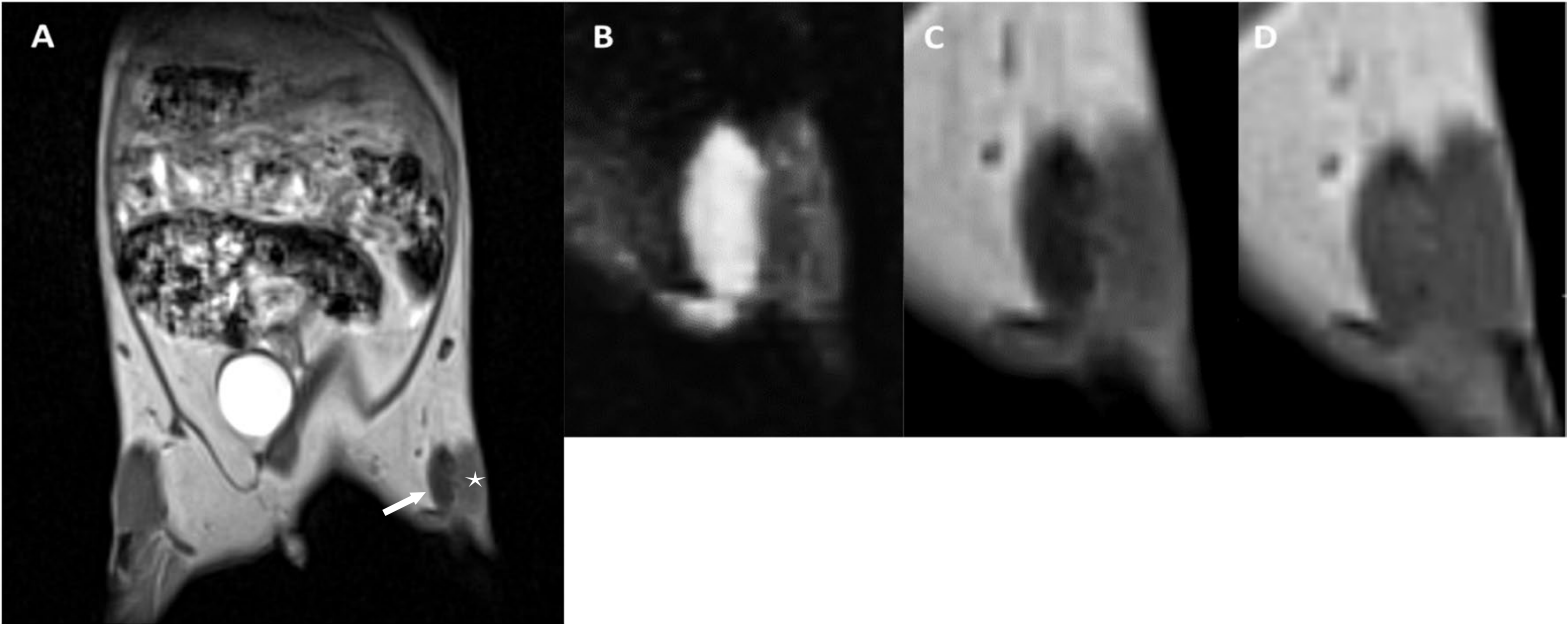
Transplant failure. **A** Coronal reconstruction of the 3D T1w volume precontrast MR-data set with a 0.3-mm slice thickness showing the abdomen, pelvis, and thighs of the mouse and the localization of the xenograft (white arrow) near a muscle bundle (white asterix). Eighty-eight days after cell transplantation, the xenograft measured 5.4 × 1.4 mm, had low signal intensity and was clearly distinguishable from the subcutaneous fatty tissue and the muscle. B—D A triplet of the zoomed xenograft is given. In contrast to the successful tumor growth in Fig. 2, the inner structure of the xenograft is homogeneous with high signal in the T2w image **B** and low signal in the T1w precontrast image **C**. A relatively homogeneous enhancement of the entire lesion could be seen after administering contrast agent **D**. No tumor cells could be detected in the histological work-up

The margins of the tumors could be seen on precontrast T1w images. The tumors were easily identified on T1w images with low SI and on T2w fat suppressed images with high SI at the site of injection and could be clearly distinguished from the subcutaneous fatty tissue (Figs. 1,2).

### Visual assessment

All H-TCP led to mass effect and architectural distortions. Delineation of a margin isointense to the neighboring muscle on precontrast T1w images (100% H-TCP vs. 40% L-TCP and 0% N-TCP) that becomes thicker with nodular solid parts over time constituted a reliable sign (the rim sign) that a cell implant would develop into an H-TCP (*p* < 0.05) (Supplementary Table S1; Fig. 1,4). Necroses and feeding vessels were only identified in H-TCP (30% and 50%, respectively) (Fig. 1). In contrast, all 6 N-TCP lesions showed a rim with a low SI in precontrast T1w without any solid components (Fig. 2,4) (*p* < 0.05).

### Quantitative longitudinal assessment

**Fig. 3:**
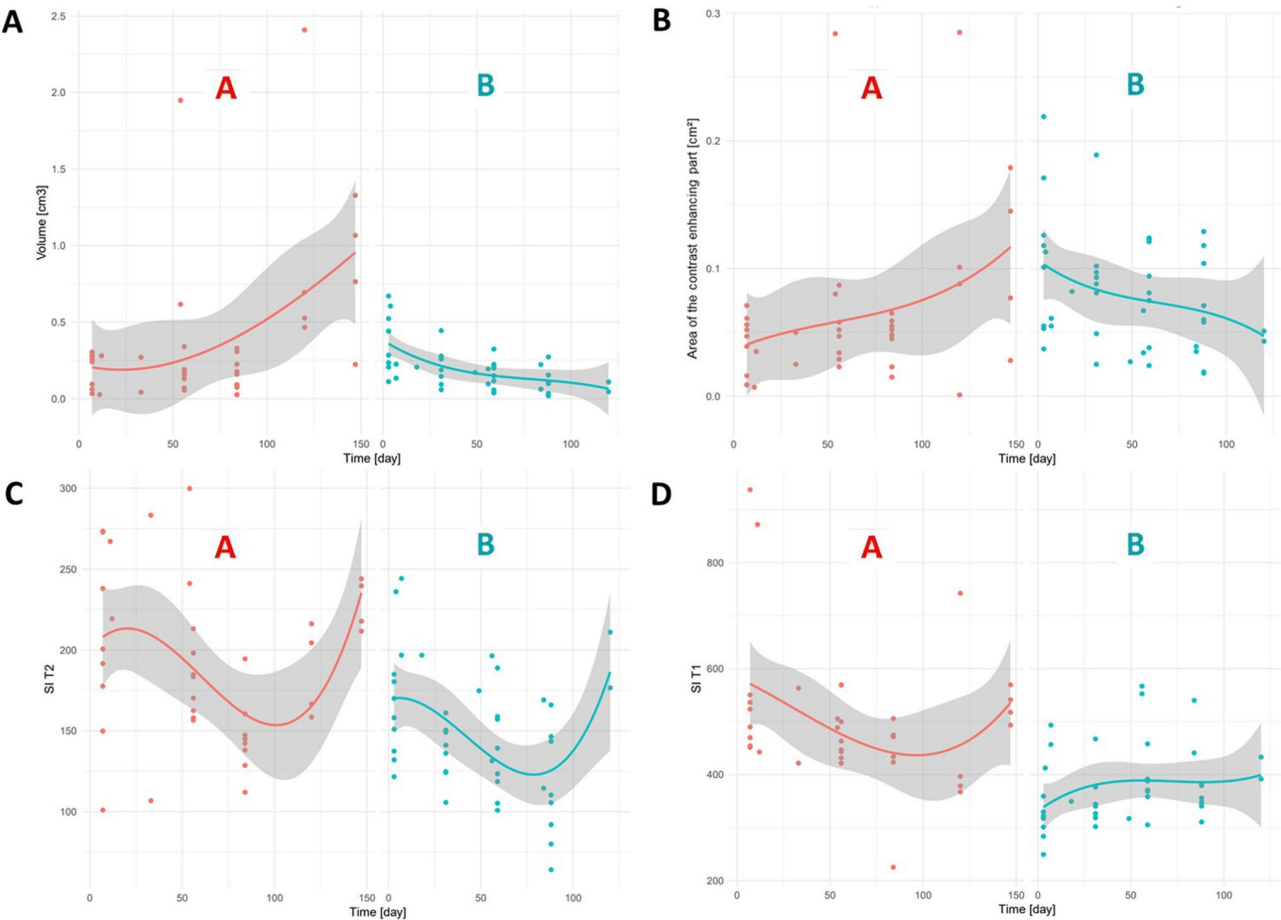
Scatter plot with fitted regression function for quantitative parameters: Volume **A**; Area of the contrast enhancing part **B**; T2w Signal Intensity **C**, and T1w Signal Intensity **D**. Group A = tumors with high tumor cell proliferation (H-TCP) and group B = tumors with low or no tumor cell proliferation (L-TCP/N-TCP). The solid, bold lines display the fitted regression line for each variable with darkened areas representing the 95% confidence interval. Note that the volume increased in H-TCP, whereas in 100% of N-TCP and 80% of L-TCP a continuous decrease in volume was observed over time (*p* < 0.05)

**Fig. 4:**
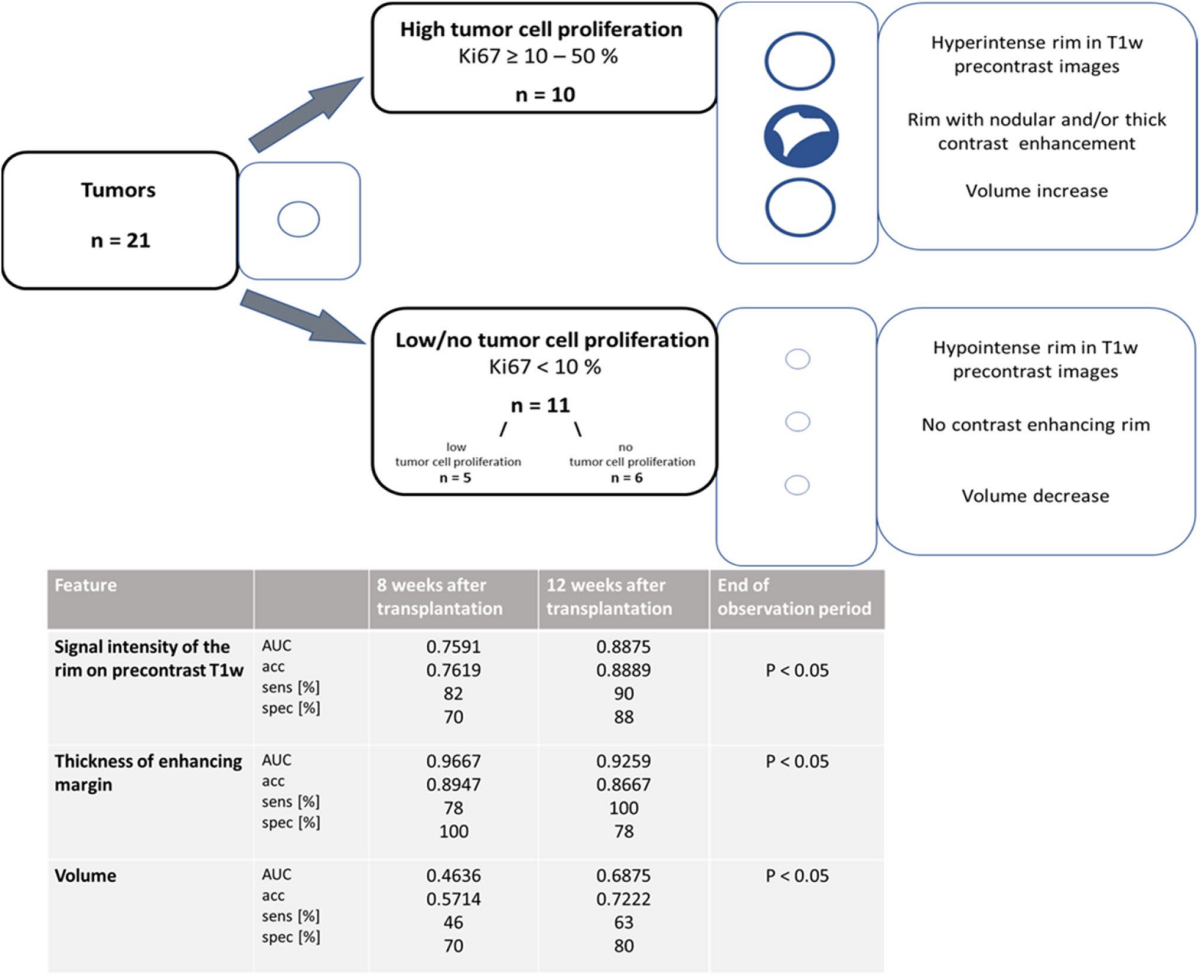
Summary of the significantly different features of qualitative and quantitative MR measurements. The 21 tumors were divided into two groups: high tumor cell proliferation (Ki67 ≥ 10%—50%), and low or no tumor cell proliferation (Ki67 < 10%). The features `Signal intensity of the rim on precontrast T1w`, `Thickness of enhancing margin`, and `Volume` turned out to be significantly different between the two groups at the end of the observation period (*p* < 0.05) (table last column). Characteristic MR lesion morphology per feature is shown schematically in the graphics for both groups. In addition, the predictor value for each of the three MR features is provided at 8 and 12 weeks after transplantation (3rd and 4th column of the table). Note: AUC = area under the curve; acc = accuracy; sens = sensitivity; spec = specificity

The volume of all tumors changed during the observation period. In 20% H-TCP, the volume increased continuously, whereas in 100% N-TCP and 80% L-TCP a continuous decrease in volume was observed over time (*p* < 0.05) (Fig. 3A,4).

In the remaining H-TCP (80%), a single dip in volume either was observed between the first and second measurement (60%) or between the second and third measurement (20%) (the individual time-volume curve for each tumor is shown for the first three MR measurements in the supplement: Supplementary Figure S4).

Any further decrease in volume during the course indicated that the tumor had low or no growth potential at all (*p* < 0.05). A decrease in volume of less than 4% occurred only in H-TCP (20%); otherwise, the extent of the first dip in volume was more pronounced but had no impact on the further course (range: H-TCP 21–59%, L-TCP 14–61%, and N-TCP 34–66%). In tumors with densely packed cells, without exceptions (100% H-TCP), volumes were higher at the end of the observation period than at the first measurement (*p* < 0.05) (Fig. 3A).

The area of the contrast enhancing part of the tumor increased in all H-TCP, although not reaching the level of significance in the temporal course (Fig. 3B). In 14/85 measurements, no signal increase in T1w could be found after administering contrast agent, which was interpreted as a failed administration of the contrast agent.

A significant difference in the temporal course of the T2w SI was detected, where all H-TCP showed a higher signal in the last measurement at the end of the observation period than in the penultimate measurement (*p* < 0.05) (summary of time point combinations by ANOVA and Tukey-Cramer statistics for signal intensity measurements are provided in the supplement: Supplementary Table S5; Fig. 3C). The temporal course of the T1w SI pre- and postcontrast did not reach the level of significance, however (**S**upplementary Table S5; Fig. 3D).

The HI did not reach the level of significance in the temporal course, either in T2w images or in T1w pre- and postcontrast images.

### Radiomic feature analysis

MaZda software extracted 314 radiomic features from the images. Feature reduction was achieved by excluding nonreproducible results through an interobserver study, by excluding unconfirmed radiomic features after applying the Boruta algorithm (an overview is given in the supplement: Supplementary Figure S6) and, finally, by selecting only one radiomic feature in cases of clustered correlations. Of the original data set with 314 radiomic features, 24 were wavelet transform-derived, with 6 of them were classified as confirmed with a positive match rate (against shadow features) of more than 80% by the Boruta algorithm. Of the remaining 290 first or second order features, 11 were of the classified as confirmed (match rate > 80%).

All confirmed radiomic features with a higher than 0.80 according to Boruta feature selection algorithm that are applied to the test data set are listed in Table 1. Wavelet transform parameters were best for distinguishing between H-TCP and L-TCP/N-TCP (AUC 0.98) and were superior to the parameters “total tumor volume” (AUC 0.92) and “contrast-enhancing part of the tumor” (AUC 0.83) (AUCs are provided in the supplement: Supplementary Figure S7

**Table 1:**
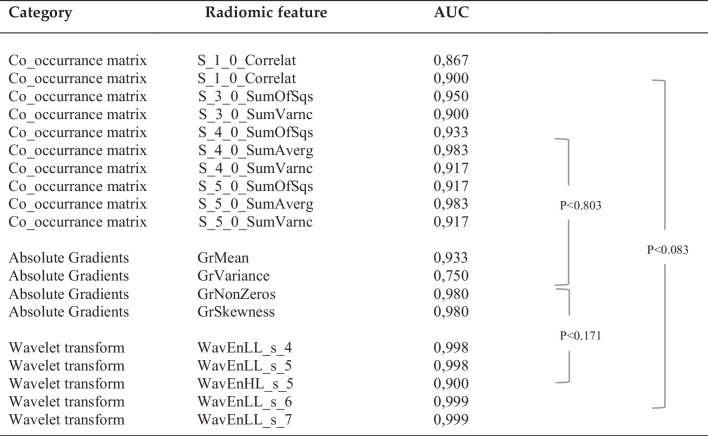
Summary of the most important radiomic features applied on the test data set according to their area under the curve (AUC) of receiver operating characteristic

Wave_LL_s7 = wavelet decomposed with low-pass filtering in two directions; Wave_HL_s5 = wavelet decomposed with high-pass filtering in one direction and low-pass filtering in the other. S_0_5_sum_variance = with S meaning summation, 0 the direction (in degrees) and 5 the distance of two pixels. S_0_4_sum_squares = with S meaning summation, 0 the direction (in degrees) and 4 the distance of two pixels

## Discussion

Increasing volumes, a rim isointense to the neighboring muscle in T1w precontrast images, progressive solid tumor components, neovascular proliferation, and necrosis in the tumor matrix represent the hallmarks of tumor growth in this study. Applying these qualitative and quantitative criteria, it can be determined at an early stage in serial examinations over time whether a heterotopically transplanted glioblastoma in mice will develop into a tumor with H-TCP. This information can be used to select animals with H-TCP before randomization in experimental therapy studies.

Hanahan and Weinberg proposed that six hallmarks of cancer together constitute an organizing principle that provides a logical framework for understanding the remarkable diversity of neoplastic diseases [26]. They include sustaining proliferative signaling, evading growth suppressors, resisting cell death, enabling replicative immortality, inducing angiogenesis, and activating invasion and metastasis. Correspondingly, we found that after an initial decrease in all tumors, the volumes of xenografts with H-TCP increased, whereas those with L-TCP or N-TCP continued to decrease over time.

Using MRI, with its superb tissue contrast and spatial resolution, the internal morphology of a tumor can be analyzed in detail. Image contrast depends on several factors, such as the intrinsic MRI properties of tissue, the acquisition strategy, and the imaging hardware. Intrinsically, the transverse and longitudinal relaxation times (T2 and T1) are related to the micro- and macrostructural characteristics, including tissue density (water content and mobility), macromolecule, protein, and lipid composition, and paramagnetic atom (e.g., iron) concentration [27]. Our visual evaluation of the tumors over time revealed that all of the histologically confirmed H-TCP showed a margin isointense to the neighboring muscle on precontrast T1w that became thicker along with increasing nodular solid parts (rim sign). This finding seems contradictory as diffuse infiltration is a well-established key feature of human glioblastoma [8]. However, the lack of tumor infiltration is known in orthotopic xenograft models of glioblastoma: depending on the cell line used, tumors can be well delimited and surrounded by reactive astrocytes without diffuse infiltration [28–31]. Generally, the high SI on precontrast T1w can be influenced by increased paramagnetic effects from prior hemorrhage (ferritin, hemosiderin, deoxyhemoglobin, and/or methemoglobin deposition) or free radicals and other nonparamagnetic effects, for example, very high (nonparamagnetic) protein concentration [32]. In tumors, additionally, the changes in SI are assumed to be the result of a low spin density due to an increasing cellularity associated with a scant cytoplasm, where the high nucleus-to-cytoplasm ratio is a strong predictor of malignancy [32, 33].

In the histopathological work up in our study, regarding the morphology, an astroglial (glioblastoma-typical) differentiation was evident. Each cell type of glial tissue as astrocytes, oligodendrocytes, microglia, and ependymal cells has its own function and may produce its own glioma [34]. According the World Health Organization (WHO) classification, glioma have different grading, which indicates the tumors’ malignancy. Glioblastoma are WHO grade IV tumors, which are characterized by increased cellularity, frequent mitoses, nuclear atypia and pleomorphism, microvascular proliferation and necrosis [35].

To measure cell proliferation in gliomas, immunohistochemical assessment of the Ki67 has become the most widely used method in the diagnostic setting [36]. Ki67 is a nuclear protein present during all active phases of the cell cycle and strictly associated with cell proliferation [37, 38]. Ki67 increases with increasing WHO grade with high-grade CNS tumors showing proliferation activities of 10% or more [39]. Based on this, in our study we required a Ki67 ≥ 10% for assignment to the successful transplant group (H-TCP).

Glioblastoma are characterized by exuberant angiogenesis, a key event in tumor growth and progression [40]. This is in line with our results: a specific sign of H-TCP in our study was the presence of neovascular proliferation with profound hypervascularity of the tumor at both the radiological and pathological examinations, where signal void from rapid flow in T2w and flow-related enhancement in tumor vessels in T1w caused changes in SI [32]. Tumor progression is often accompanied by ingrowth of blood vessels either through vessel co-option of the pre-existing vasculature [41] or by inducing new blood vessel formation through a variety of molecular and cellular mechanisms [42]. One mechanism may involve cooption of native blood vessels by glioma cells inducing expression of angiopoietin-2 by endothelial cells. Subsequently, vascular apoptosis and involution leads to necrosis and hypoxia. This in turn induces angiogenesis that is associated with expression of hypoxia-inducible factor (HIF)-1α and vascular endothelial growth factor (VEGF) in perinecrotic pseudopalisading glioma cells [40]. The degree of angiogenesis represents a direct correlate of tumor grade in human glioma. For example, tissue factor, the catalyst of the extrinsic pathway of hemostasis, is overexpressed under hypoxic conditions and could be involved in vascular thrombosis. Thus, vascular regression and necrosis constitute necessary events for the subsequent development of angiogenesis [40, 43–46].

Necrosis has also been consistently used as a grading criterion in gliomas [40, 47] and is typically observed in glioblastoma [40, 47, 48]. Accordingly, necrosis is a further specific finding that was only exhibited in H-TCP at both the radiological and histological examinations in our study. On MRI necrosis may present either with high SI or low SI on both T1w and T2w images, depending on the presence of naturally occurring paramagnetic cations and free radicals [27]. These substances usually shorten relaxation times, whereas regions of cystic necrosis prolong relaxation times [49]. Dean et al. evaluated the characteristics for classifying gliomas by MRI imaging and found mass effect, cyst formation, and necrosis to be positive predictors of malignancy [50]. In our study, the MRI features of the gliomas with H-TCP are mirrored by the histological findings as sites of necrosis. Importantly, it was not the extent of the contrast-enhancing parts of the tumor that correlated with the cellularity of the xenograft, but rather the thickness of the rim and the extent of the solid nodular contrast-enhancing parts, some of which were located in the rim. These findings are crucial when assessing postcontrast images to determine the effect of therapy in clinical trials.

Although we could show that conventional analysis of the internal structure of a tumor can accurately distinguish tumors with a high tumor cell proliferation from those with low or no tumor cell proliferation, automated radiomic feature analysis offers the potential to overcome the limitations of a purely visual image interpretation (interobserver variability, qualitative evaluation). Radiomic feature analysis supplies a large range of techniques for quantifying gray-level patterns and pixel inter-relationships within an image, providing a measure of heterogeneity. The wavelet transform technique analyzes the frequency content of an image within different scales and frequency directions. In our study, two features derived from radiomic feature analysis – both members of the wavelet transform family—showed the best discrimination between H-TCP and L-TCP/N-TCP.

Prerequisites for obtaining high tissue contrast with MRI are a sophisticated acquisition strategy and dedicated imaging hardware. The in vivo imaging of small animals undoubtedly represents one of MR microscopy’s most valuable capabilities [5]. In our study we used a clinical 3 T body scanner with a dedicated small-animal coil and an MRI-compatible, low-cost solution to monitor the respiration and heart rate of mice and with an effective respiratory trigger system for suppressing motion artifacts as described by Herrmann et al. [4]. Using this setting, Herrmann et al. stated that an impressive abdominal image quality can be achieved with an isotropic spatial resolution of (0.33 mm)^3^ within short scan times. By increasing the scan time to 30 – 60 min, even resolutions of (0.2 mm)^3^ can be achieved with sufficient image quality. In our study, the acquisition time of the 3D T1w VIBE sequence was 8 min, 44 s and 3 s longer for the 3D T2w SPACE. We achieved a voxel size of 0.26 × 0.26 × 0.25 mm (T1w VIBE) or 0.24 × 0.24 × 0.25 mm (T2w SPACE), which provides an excellent histological-radiological correlation.

### Limitations

This study has some limitations. Firstly, the method does not reflect the tumor microenvironment, where interactions between glioma cells and the surrounding brain tissue are complex. In addition, it does not reproduce intracerebral infiltrative tumor growth behavior in glioblastomas. Therefore, applying an orthotopic xenograft model would have given the study more strength. However, the observation period in such an experimental setting would have been significantly shorter due to anatomical restrictions and associated with higher stress and higher mortality for the test animals. Secondly, the number of mice that could be included was small. Including a larger number of animals would have increased confidence in the conclusions of this study. On the other hand, the strength of this study is that all mice were examined by strictly following a standardized MRI protocol over a long follow-up period with clear inclusion and diagnostic criteria and was validated against histopathological findings, thus forming a homogeneous group. Thirdly, when a large number of radiomic variables are applied to a small number of observations, such as the number of animals in our study, there is a high risk of overfitting the model. Therefore, our results concerning the analysis of radiomic parameters must be interpreted with great caution and ideally verified on a much larger number of observations. Finally, we did not compare the results of morphological MRI with advanced functional and metabolic MRI or with other imaging modalities, which could have put the usefulness of our method into a larger perspective.

## Conclusions

We showed that visual and texture analysis-based assessment of the internal structure of heterotopically implanted glioblastomas makes it possible to distinguish between xenografts with high tumor cell proliferation and xenografts with low or no tumor cell proliferation. Furthermore, it can be determined at an early stage whether a given xenograft will develop into a tumor with high tumor cell proliferation. In contrast to the method of measuring the total tumor volume alone to monitor tumor growth, our approach provides reproducible and quantifiable results to determine the cell proliferation of a tumor, which is an important parameter of any therapy study intended to show the effect of tumor suppression.

Finally, the present study shows that, by inexpensively supplementing existing technical equipment, this examination technique can be made broadly available and generate relevant information on the intrinsic tissue characteristics down to a slice thickness of 0.25 mm, thus contributing to translational research.

### Supplementary Information

Below is the link to the electronic supplementary material.

Supplementary file1 (DOCX 3.16 MB)

## Data Availability

The data presented in this study are available on request from the corresponding author.

## Abbreviations

CE: contrast enhanced
Gd DTPA: gadolinium Diethylenetriaminepentaacetic acid
GFAP: glial fibrillary acidic protein
HI: heterogeneity index
H-TCP: high tumor cell proliferation
L-TCP: low tumor cell proliferation
MR: magnetic resonance
MRI: magnetic resonance imaging
N-TCP: no tumor cell proliferation
ROI: region of interest
SI: signal intensity
SPACE: Sampling Perfection with Application optimized Contrasts using different flip angle Evolution
T: Tesla
T1w: T1-weighted
T2w: T2-weighted
VASARI: Visually AcceSABLE Rembrandt Images
VIBE: Volumetric interpolated breath-hold examination
