# Supplementary material for: Radiomics and visual analysis for predicting success of transplantation of heterotopic glioblastoma in mice with MRI
Source: J Neurooncol. 2024 Jul 3;169(2):257–67. doi: 10.1007/s11060-024-04725-z (PMC11341603; doi:10.1007/s11060-024-04725-z)
Supplement: Supplementary file 1 — Supplementary file1 (DOCX 3.16 MB) [file 11060_2024_4725_MOESM1_ESM.docx]

**Supplement**

**Supplementary Table S1****.** Overview of the results of visual qualitative analysis.

| **Feature** | **Description** | **Score** | **Ki67**  **≥ 10% < 10%**  **n=10 n=11** | |
| --- | --- | --- | --- | --- |
| Enhancement Quality | Qualitative degree of contrast enhancement is defined as having all or portions of the tumor that demonstrate higher signal on the postcontrast T1w images compared to precontrast T1w images. | 0= n/a  1= None  2= Mild  3= Marked | | 2 2  0 0  4 5  4 4 |
| Proportion Enhancing | The proportion of the entire tumor that is enhancing. | 0= n/a  1= None  2= <1/3  3= 1/3-2/3  4= >2/3  5= All | | 2 2  0 0  0 0  1 1  7 2  0 6 |
| Proportion Necrosis | Necrosis is defined as a region within the tumor that does not enhance or shows markedly diminished enhancement, is hyperintense on T2w images, is low on T1w images, and has an irregular border. | 0= n/a  1= None  2= <1/3  3= 1/3-2/3  4= >2/3  5= All | | 0 0  7 11  0 0  2 0  1 0  0 0 |
| Cyst(s) | Cysts are well-defined, rounded, often eccentric regions of very bright T2w signal and low T1w signal, with very thin, regular, smooth, nonenhancing or regularly enhancing walls, possibly with thin, regular, internal septations. | 0= n/a  1= No  2= Yes | | 0 0  10 11  0 0 |
| Signal intensity of the rim on precontrast T1w | If the SI of the rim is equal to the SI of the neighboring muscle in T1w precontrast images, then the scoring is isointense; if lower it is hypointense. | 0= n/a  1= isointense  2= hypointense | | 0 0  10 2  0 9 |
| Thickness of enhancing margin | The enhancing margin score is not applicable if there is no contrast enhancement. If most of the enhancing rim is thin, regular, and has homogeneous enhancement, the grade is ‘thin’. If most of the rim demonstrates nodular and/or thick enhancement, the grade is ‘thick’. If there is only enhancement and no rim, the grade is ‘none‘. | 0= n/a  1= None  2= Thin  3= Thick | | 2 2  0 7  0 1  8 1 |
| Definition of the enhancing margin | The scoring is not applicable if there is no contrast enhancement. Assess whether most of the outside margin of the enhancement is well defined or poorly defined. | 0= n/a  1= None  2= Well-defined  3= Poorly-defined | | 0 0  0 0  10 11  0 0 |
| Hemorrhage | Intrinsic hemorrhage in the tumor matrix. Any intrinsic foci of low signal on T2w or high signal on T1w images. | 0= n/a  1= No  2= Yes | | 0 0  10 11  0 0 |
| Neovascular proliferation | Profound hypervascularity of the tumor with formation of large vessels. Linear or serpentine regions of signal void within and about the tumor. | 0= n/a  1= No  2= Yes | | 0 0  5 11  5 0 |

Note. Column 4 shows the results of the last MR examination immediately before euthanasia of the mice for histological workup for each tumor: The 21 tumors were divided into two groups based on their immunocytochemistry for Ki67. Abbreviations: n/a = not applicable; SI = Signal Intensity.

**Supplementary Table S2****.** List of radiomic features provided by the MaZda software.

| **Category** | **Radiomic features** |
| --- | --- |
| Histogram | Mean, Variance, Skewness, Kurtosis, Percentage 0.1%, Percentage 10%, Percentage 50%, Percentage 90%, Percentage 99% |
| Absolute gradient | Mean, Variance, Skewness, Kurtosis, Percentage of pixels with nonzero gradient |
| Run-length matrix | Run length nonuniformity, Gray level nonuniformity, Long-run emphasis, Short-run emphasis, Fraction of image in run |
| Co-occurrence matrix | Angular second moment, Contrast, Correlation, Sum of squares, Inverse difference moment, Sum average, Sum Variance, Sum entropy, Entropy, Difference variance, Difference entropy |
| Autoregressive model | Theta 1-4, Sigma |
| Wavelet transform | High-pass filtering in both directions, High-pass filtering/Low-pass filtering, Low-pass filtering/High-pass filtering, Low-pass filtering in both directions |


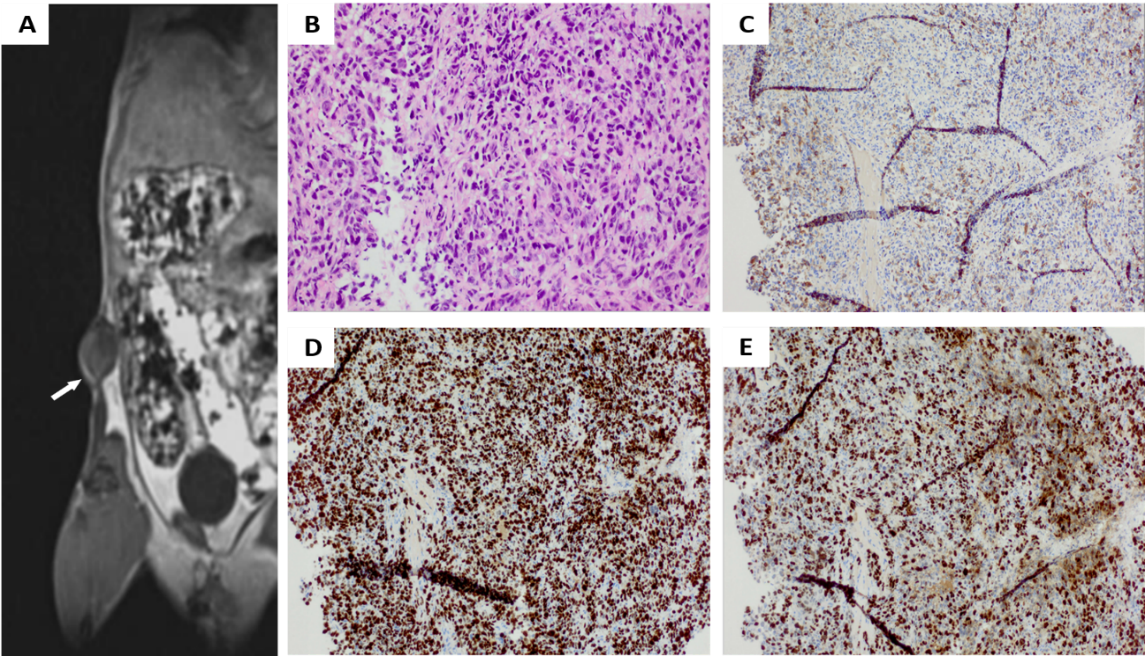


**Supplementary Figure S3****.** Example of successful tumor growth and histological work-up. **A** Coronal reconstruction from the 3D volume T1w MRI-data set with 0.3-mm slice thickness showing the abdomen, pelvis, and thigh of the mouse. The tumor in the left groin had a maximum diameter of 4.7 × 3.2 mm (white arrow). B-E Histological examination revealed a high density of pleomorphic cells interspersed with endothelial cell-proliferating neovascularization. At higher magnification **B**, stained with H&E, neoplastic cells showed highly nuclear polymorphisms of imposing glial cells and frequent mitotic figures. Immunohistochemistry revealed positive staining for glial fibrillary acidic protein (GFAP) **C** and for MIB-1 **D** in the tumor cells and a nuclear accumulation of p53 **E**

**
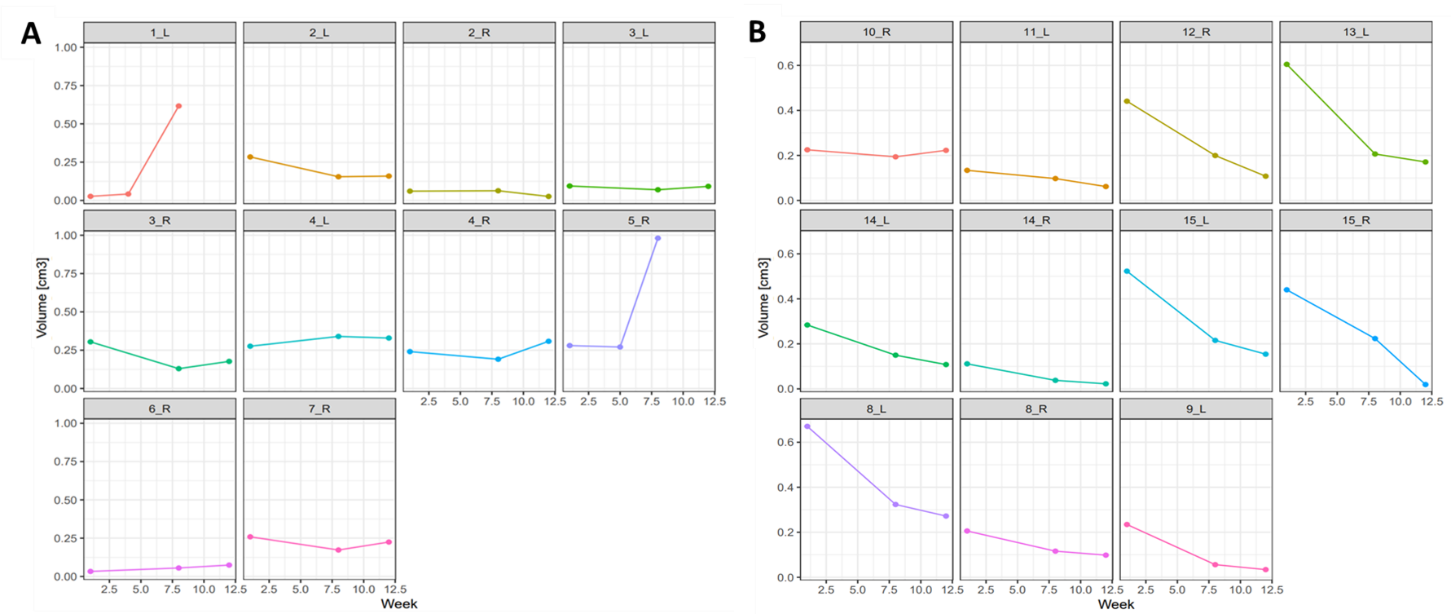
**

**Supplementary Figure S4.** Time-volume curves for each tumor in the early phase after transplantation. The individual time-volume curve for each tumor is shown for the first three MR measurements: directly after the transplant and the other two measurements 8 and up to 12 weeks after the transplant. The heading of each diagram shows the mouse number and the side of implantation (R = right; L = left). In tumors with high tumor cell proliferation **A**, the volume increased continuously or with a single dip at the second measurement. In contrast, all tumors with low or no tumor cell proliferation **B**, with one exception, showed a continuous decrease in volume

**Supplementary Table** **S5.** Summary of time point combinations by ANOVA and Tukey-Cramer statistics for signal intensity measurements.

| **Pair of**  **time points** | **CE_T1w_SI** | **T1w_SI** | **T2w_SI** |
| --- | --- | --- | --- |
|  | **p adj** | **p adj** | **p adj** |
| 1B - 1A | 0.9999998 | 0.3217124 | 0.9999866 |
| 2A - 1A | 0.9999805 | 0.4803694 | 0.9998999 |
| 2B - 1A | 0.9979180 | **0.0015668*** | 0.9999695 |
| 3A - 1A | 0.9892733 | 0.0784794 | 0.9939779 |
| 3B - 1A | 0.9999843 | 0.0012924 | 0.9962934 |
| 4A - 1A | 0.9991760 | 0.8103599 | 0.9674461 |
| 4B - 1A | 0.9998988 | **0.0001741*** | 0.9867009 |
| 5A - 1A | 0.9996932 | 0.9709153 | 0.1632562 |
| 2A - 1B | 0.9999914 | 0.0506395 | 0.9999104 |
| 2B - 1B | 0.9974715 | 0.9777955 | 0.9959214 |
| 3A - 1B | 0.9851000 | 0.3860826 | 0.9375942 |
| 3B - 1B | 0.9999933 | 0.9841907 | 0.9503547 |
| 4A - 1B | 0.9992233 | 0.0279154 | 0.9965877 |
| 4B - 1B | 0.9898988 | 0.9999964 | 0.9994178 |
| 5A - 1B | 0.9997725 | 0.1127340 | 0.2689050 |
| 2B - 2A | 0.9999898 | 0.4473999 | 0.9999968 |
| 3A - 2A | 0.9995898 | 0.9912736 | 0.9974969 |
| 3B - 2A | 0.9998878 | 0.4123938 | 0.9986268 |
| 4A - 2A | 0.9999977 | 0.9999808 | 0.9472767 |
| 4B - 2A | 0.9999844 | 0.1318405 | 0.9749521 |
| 5A - 2A | 0.9999994 | 0.9999204 | 0.1371959 |
| 3A - 2B | 0.9999995 | 0.9514704 | 0.9999328 |
| 3B - 2B | 0.9999704 | 0.9998999 | 0.9999809 |
| 4A - 2B | 0.9898998 | 0.2843035 | 0.8272279 |
| 4B - 2B | 0.9972366 | 0.9984074 | 0.8851199 |
| 5A - 2B | 0.9988999 | 0.4850031 | 0.0712756 |
| 3B - 3A | 0.9991343 | 0.9378279 | 0.9999999 |
| 4A - 3A | 0.9999995 | 0.9389757 | 0.5819650 |
| 4B - 3A | 0.9850583 | 0.6263909 | 0.6508105 |
| 5A - 3A | 0.9999997 | 0.9600180 | **0.0313482*** |
| 4A - 3B | 0.9999935 | 0.2583824 | 0.6054201 |
| 4B - 3B | 0.9999874 | 0.9991003 | 0.6748635 |
| 5A -3 B | 0.9999985 | 0.4579983 | **0.0327434*** |
| 4B - 4A | 0.9990673 | 0.0746638 | 0.9999999 |
| 5A - 4A | 0.9999999 | 0.9998888 | 0.7215315 |
| 5A - 4B | 0.9997028 | 0.2056103 | 0.5714698 |

Note. The first column shows all combinations of time points for MR measurement (numeric, 1-5) and the two groups of tumors with high tumor cell proliferation (A) versus tumors with low or no tumor cell proliferation (B). Columns 2 to 4 show the adjusted p-values derived from the pairs for CE_T1w_SI, T1w_SI, and T2w_SI. CE_T1w_SI: Signal intensity of the whole tumor area after administering contrast agent. T1w_SI: Signal intensity of the whole tumor area on precontrast T1w images. T2w_SI: Signal intensity of the whole tumor area on T2w images. P-values are adjusted for multiple testing; Bold numbers with *: significant difference with level of significance set at 0.05.


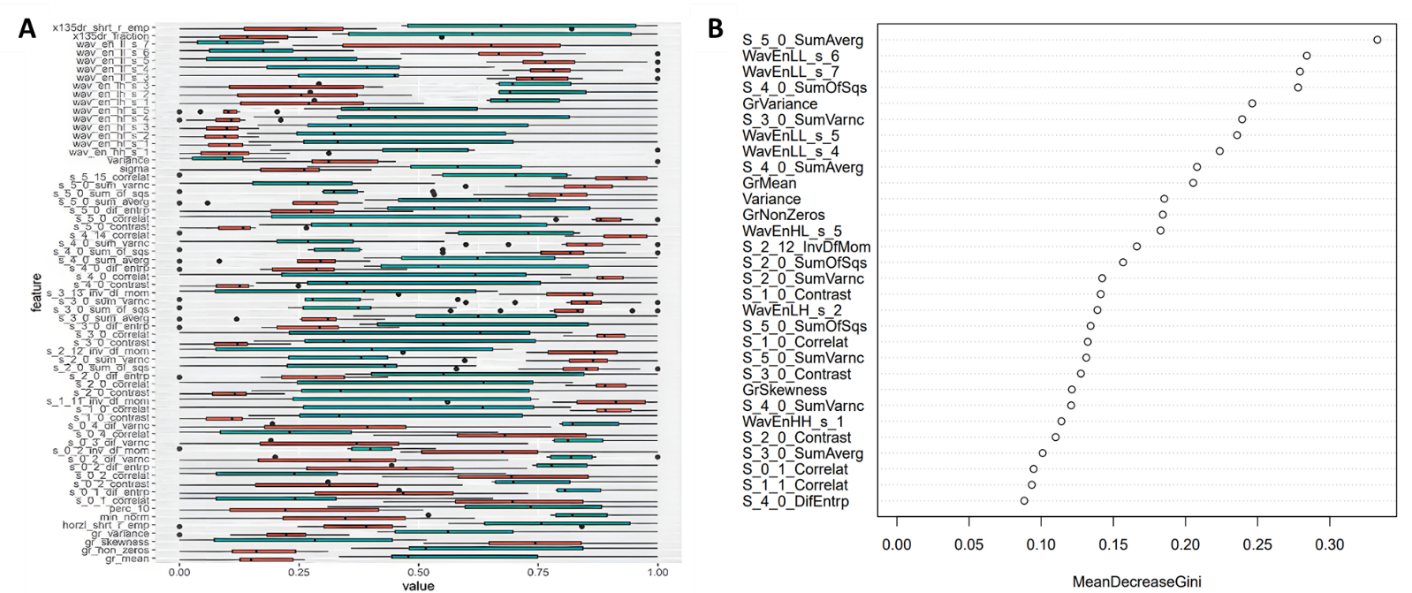


**Supplementary Figure S6.** Feature selection using Boruta algorithm. **A** Boxplot showing the normalized values of all radiomic features confirmed by the Boruta algorithm with scores higher than 0.48 after 99 iterations. Note: Group A in red = tumors with high tumor cell proliferation (H-TCP); group B in green = tumors with low or no tumor cell proliferation (L-TCP/N-TCP). **B** Mean decrease Gini score of the confirmed features. The horizontal coordinate and vertical coordinate independently represent feature importance and feature names. The variables are presented in descending order of importance, with highest in the upper right corner


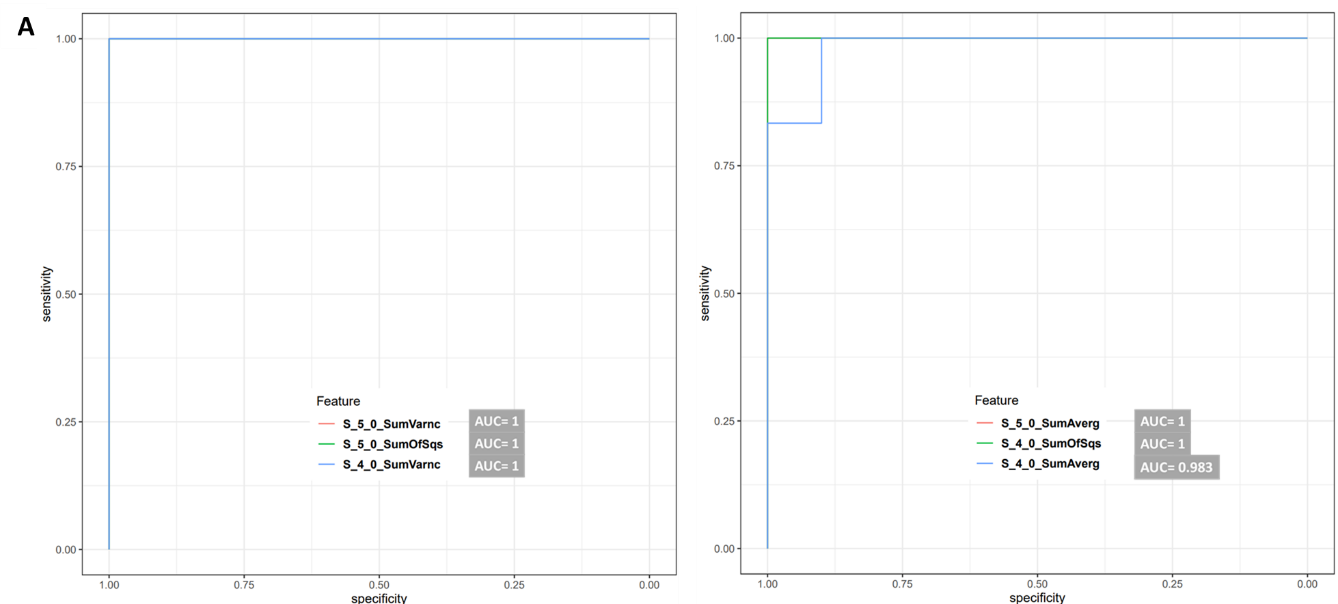


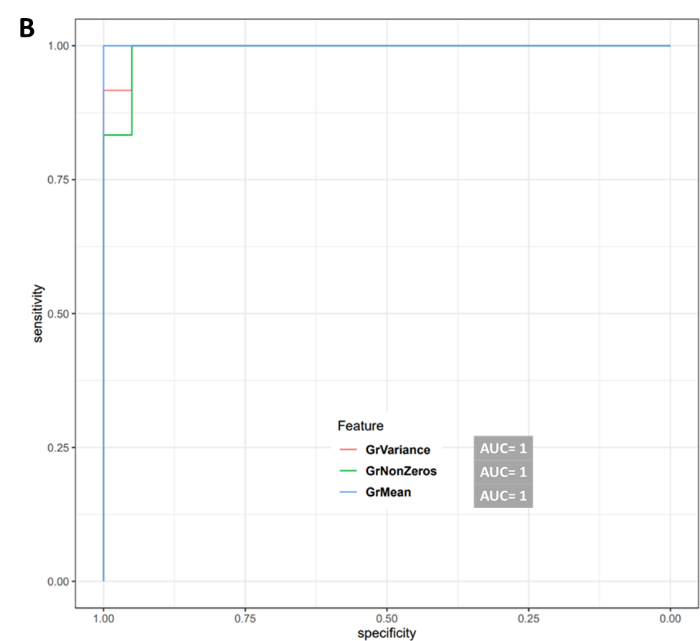


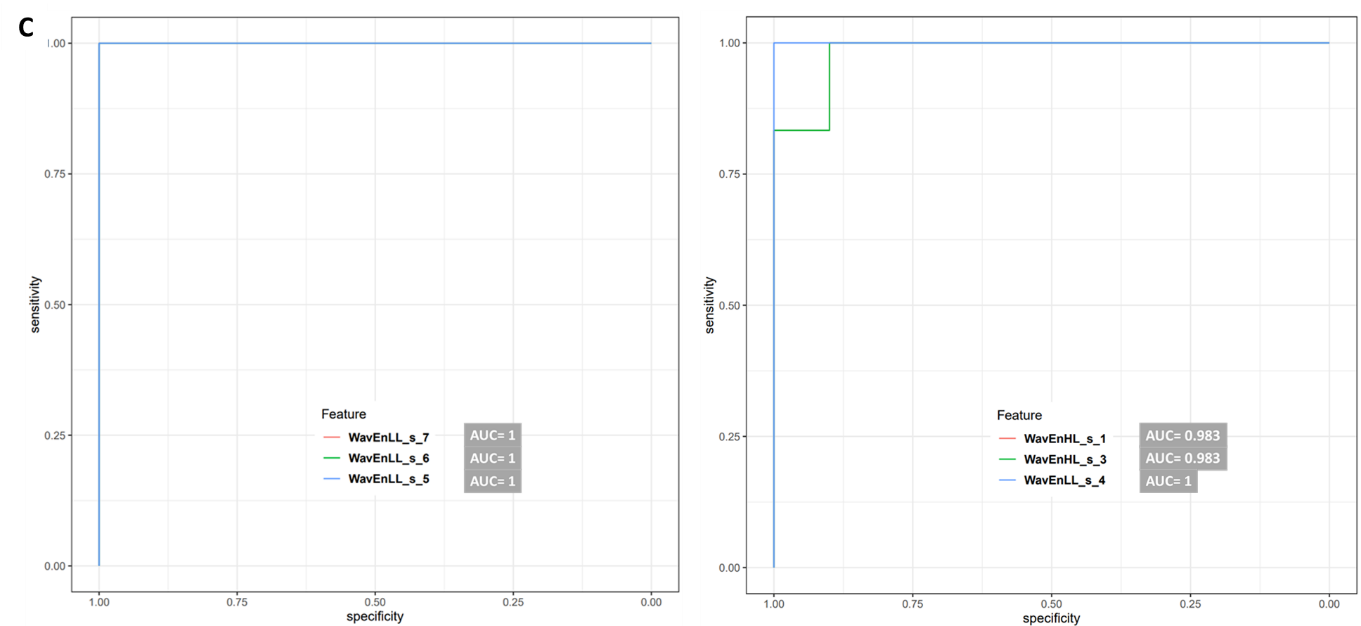


**Supplementary Figure S7:** AUCs of the radiomic features used for the test data set. Area under the Receiver Operating Characteristic (ROC) curve is shown for the best performing radiomic features of each category: **A** Co_occurrance matrix, **B** Absolute Gradients, and **C** Wavelet transform
